# Pattern of risks of rheumatoid arthritis among patients using statins: A cohort study with the clinical practice research datalink

**DOI:** 10.1371/journal.pone.0193297

**Published:** 2018-02-23

**Authors:** Hilda J. I. de Jong, Jan Willem Cohen Tervaert, Arief Lalmohamed, Frank de Vries, Rob J. Vandebriel, Henk van Loveren, Olaf H. Klungel, Tjeerd P. van Staa

**Affiliations:** 1 Centre for Health Protection, National Institute for Public Health and the Environment, Bilthoven, The Netherlands; 2 Department of Toxicogenomics, Maastricht University Medical Center, Maastricht, The Netherlands; 3 Division of Pharmacoepidemiology and Clinical Pharmacology, Department of Pharmaceutical Sciences, Faculty of Sciences, Utrecht Institute for Pharmaceutical Sciences, Utrecht University, Utrecht, The Netherlands; 4 Clinical and Experimental Immunology, Maastricht University, Maastricht, The Netherlands; 5 Sint Franciscus Gasthuis, Rotterdam, The Netherlands; 6 Department of Clinical Pharmacy, University Medical Center Utrecht, Utrecht, The Netherlands; 7 Department of Clinical Pharmacy & Toxicology, Maastricht University Medical Center, Maastricht, The Netherlands; 8 Care and Public Health Research Institute (CAPHRI), Maastricht University, Maastricht, the Netherlands; 9 Department of Non-communicable Diseases, London School of Hygiene & Tropical Medicine, London, United Kingdom; 10 Health eResearch Centre, Farr Institute for Health Informatics Research, University of Manchester, Manchester, United Kingdom; China Medical University, TAIWAN

## Abstract

We examined the association between statin use and the risk of rheumatoid arthritis (RA), with special focus on describing the patterns of risks of RA during statin exposure in a large population-based cohort in the United Kingdom. In the Clinical Practice Research Datalink, patients aged ≥40 years with at least one prescription of statins (1995–2009) were selected, and matched by age (+/-5 years), sex, practice and date of first prescription of statins to non-users. The follow-up period of statin use was divided into periods of current, recent and past exposure, with patients moving between these three exposure categories over time. Time-dependent Cox models were used to derive hazard ratios (HRs) of RA, adjusted for disease history and previous drug use. The study population included 1,023,240 patients, of whom 511,620 were statin users. No associations were found between RA and current (HR_adj_,1.06;99%CI:0.88–1.27) or past statin users (HR_adj_,1.18;99%CI:0.88–1.57). However, in patients who currently used statins, hazard rates were increased shortly after the first prescription of statins and then gradually decreased to baseline level. The risk of developing RA was increased in recent statin users, as compared to non-users (HR_adj_,1.39;99%CI:1.01–1.90). The risk of RA is substantially increased in the first year after the start of statins and then diminishes to baseline level. These findings may suggest that statins might accelerate disease onset in patients susceptible to develop RA, but in other patients, statins are probably safe and well tolerated, even after prolonged use. Alternatively, we cannot rule out that confounding by cardiovascular risk factors and ascertainment bias may have influenced the findings.

## Introduction

Statins (3-hydroxy-3-methylglutaryl coenzyme A reductase inhibitors) are widely prescribed drugs to reduce the risk of cardiovascular morbidity and mortality [1]. Statins exert, next to their well-known cholesterol-lowering activity, anti-inflammatory and immunomodulatory effects, and may be beneficial in the treatment of immune-mediated disorders other than atherosclerosis. Indeed, beneficial effects of statins were observed in clinical trials and experimental models of RA [2–4]. The anti-inflammatory effects of statins have been studied in several clinical trials by measuring C-reactive protein (CRP) [5,6]. In these clinical trials it has been shown that statins decrease levels of CRP [5,6]. Since statin therapy reduces the incidence of acute and chronic rejection in heart and renal transplant patients [7,8], the immunomodulating effects have been further studied. Statins have been reported to suppress interferon-γ (IFN-γ)-inducible expression of major histocompatibility complex (MHC) class II proteins in endothelial cells, monocytes/macrophages and T cells [9,10]. Another beneficial effect of statins is the effect on the T helper cells (Th1)/Th2 balance by inducing the secretion of Th2 cytokines (IL-4, IL-5 and IL-10) and transforming factor β (TGF-β), or by suppressing secretion of Th1 cytokines (IL-2, IL-12, IFN-γ and TNF-α) [11,12]. Recently, it has been suggested that statins skew T cell differentiation towards regulatory T cell (T reg) and away from pro-inflammatory Th17 cells via geranylgeranylation of proteins, resulting in promoting Treg differentiation in the periphery, while blocking Th17 cell differentiation [13]. Statins can also down-regulate expression of the co-stimulatory molecule CD40 in various cell types, e.g., endothelial cells, smooth muscle cells and macrophages [14]. Interestingly, certain types of statins selectively block the β-2 integrin, leukocyte function antigen-1 (LFA-1), thereby blocking binding to intercellular adhesion molecule-1 (ICAM-1) and thus reducing T cell activation [15].

The immunomodulating effects may, on the other hand, facilitate the development of autoimmunity potentially resulting in autoimmune diseases, such as RA [16–21]. It has been suggested that statins induce a shift from a Th1 to Th2 immune response by their direct effect on T cells. Promoting a shift from Th1 to Th2 immune responses may dysregulate the immune homeostasis and can lead to the breakdown of self-tolerance, precipitating autoimmunity [16,22]. In addition, statins are potent pro-apoptotic agents and may trigger or exacerbate cellular apoptosis [23], thereby releasing nuclear antigens into the circulation, which may foster the production of pathogenic autoantibodies [24].

Apart from our studies [17–19,21,25], six other studies have described the risk of developing RA or connective tissue disease (CTD) (the majority of the patients were coded with RA) during statin treatment, and have shown conflicting results [26–31]. Possible explanations for these conflicting findings may be attributed to the lag-time between statin use and incident RA [27,29], using different definitions of exposure to statins [19,26–31], using different definitions of RA [19,27–31], comparing to a control group of non-persistent statin users [27] or lowest duration weighted average statin intensity [31] instead of non-users, controlling for other confounders [19,26–31], shifting the date of incident RA [19,26], propensity score matching on baseline characteristics [30], or conducting separate analyses in patients with or without a medical history of cardiovascular risk factors (S1 Table) [19,26].

Cardiovascular risk factors, including smoking and hormone replacement therapy have been associated with RA [32–36]. Several studies have demonstrated an unfavourable lipid profile before a patient is diagnosed with RA [32,37]. If statin use is a proxy for hyperlipidaemia then the increased risk of developing RA in our previous study [19] might be explained by hyperlipidaemia rather than by an immunomodulating effect of statins. Otherwise, subclinical RA may have been present before the initiation of statin treatment since it is well-known that autoantibodies and non-specific symptoms may be present long before patients are diagnosed as having RA [38].

At present, the previous observational studies have not shown a conclusive relationship between statin use and the risk of developing RA. It is unclear whether the association between statin use and the risk of developing RA is related to the use of statins or whether it is merely an association with hyperlipidaemia. Moreover, none of the previous studies [19,26–31] studied the pattern of risks of RA with changes in statin exposure over time. Therefore, we examined the association between the use of statins and the risk of developing RA, with special focus on describing the patterns of risks of RA with changes in statin exposure over time and confounding by cardiovascular co-morbidities in a large population-based cohort.

## Materials and methods

### Data source

Data were derived from the Clinical Practice Research Datalink (CPRD), previously known as the General Practice Research Database, which contains computerised medical records of all patients under the care of 625 general practices (GPs) in the United Kingdom, representing 8% of the population. The CPRD has been described in detail elsewhere [39–40]. The database provides detailed information on demographics, diagnoses, prescription details, preventive care provided, specialist referrals, and hospital admissions [40]. Several independent validation studies have shown that the CPRD database has a high level of completeness and validity [41,42].

The CPRD Group has obtained ethical approval from a National Research Ethics Service Committee (NRES) for all purely observational research using anonymised CPRD data; namely, studies which do not include patient involvement (which is the vast majority of CPRD studies). Independent Scientific Advisory Committee (ISAC) (https://www.cprd.com/ISAC/members.asp) is responsible for reviewing protocols for scientific quality, but may recommend that study-specific ethical approval is sought if ethical issues arise in relation to an individual study. For the present study a separate ethical approval was not required since the patients were not directly involved in formulating the research question, nor were patients actively involved in the design and/or conduct of the research (https://www.cprd.com/isac/otherinfo.asp).

### Study population

We conducted a matched cohort study with prospectively collected data which has been described previously [25]. All patients who had at least one prescription of statins at least one year after the start of data collection (period: 1995–2009) were included. The date of the first prescription of statins was defined as the index date. Statin users were matched by age (+/-5 years), sex, and practice to a single control (non-users of statins), with the index date of the control being the same as that of the statin user (i.e. matching on calendar time). After using a matched random sampling approach, statin users and non-users who were younger than 40 years, had ever been diagnosed with RA, and/or had used disease modifying anti-rheumatic drugs (DMARDs) before or at index date were excluded.

### Exposure to statins

Exposure to statins was determined by all prescriptions, and each prescription length was calculated by dividing the number of prescribed tablets by the number of days prescribed daily dose. Compliance to statins declines substantially over time [43], and therefore the time of follow-up was divided into periods of current, recent and past exposure to statins, with patients moving between these three exposure categories over time [25]. Current exposure was defined as the days from the start date of a prescription until the start date of the consecutive prescription for statins. When the start date of the consecutive prescription of statins was prescribed within these 3 months after the start date of the prescription, patients continued to be ‘current users’. The expected duration between consecutive prescriptions was defined as 3 months, which reflects the prescribing regimen for long-term use of statins. Since patients can move between different categories of exposure to statins over time, patients can be defined more than once as ‘current users’ [25]. The duration of current statin use was calculated by estimating the number of days within each category of ‘current users’, and then the days per category were added up. We divided the duration of statin use into ≤1 and >1 year use.

We believe that ‘current users’ who initially started their statin therapy have another risk profile than ‘current users’ who restarted their therapy; therefore, ‘current users’ were divided into ‘de novo’ and ‘restart’ users. ‘De novo’ statin users were defined as patients who were not moving between the three periods of statin exposure over time. Recent exposure was defined as the period of time from 3 to 12 months after the end date of the most recent prescription, and past exposure was the period of time from 12 months or longer after the end date of the most recent prescription of statins. Three examples of time-dependent exposure to statins were illustrated elsewhere [25].

When the event (RA) occurred in a category of ‘current users’, the patient was defined at the time of the event as a current user. If not, the statin user was a recent user or a past user at the time of occurrence of the event.

### Clinical outcome

Each patient was followed from the index date up to the date of the first diagnosis of RA (identified from the CPRD Read codes), or the date when the patient left the general practice, died, or the end date of data collection, whichever date came first. Patients were considered as having a diagnosis of RA if the first diagnosis of RA registered by a GP was verified by at least one prescription of a DMARD during follow-up, adapted from an algorithm proposed by Thomas et al [44]. When a patient was previously referred to a rheumatologist, the date of the first referral was defined as the event date.

We carried out four sensitivity analyses to evaluate the impact of potential case misclassification and we changed the definition of RA:

1. into patients with a first diagnosis of RA with a referral to a rheumatologist or with at least one prescription of a DMARD;
2. into the first diagnosis of RA with a referral to a rheumatologist, or at least one prescription of a DMARD and/or at least two prescriptions of corticosteroids, a definition we used in a previous study [19];
3. into having another medical record of RA after the first diagnosis as proposed by Kim et al [45].
4. into patients with a medical record of RA who were treated with at least one prescription of a DMARD within a time span of two years after their first-time diagnosis, limiting bias due to “peeking” into the future to define RA.

In another sensitivity analysis, a lag time between the onset of RA and the diagnosis was considered [27,29], and therefore we excluded the first year of every patient following the initiation of statin treatment, thereby excluding the events of RA (S1 Fig) In addition, we examined the effect of potential late manifestation of the clinically apparent symptoms of RA by changing the event date of RA exactly one year before the first diagnosis of RA, as has been suggested by Jick et al. (S1 Fig) [26]. We studied the effect of setting the event date of RA when the date of referral to a rheumatologist was more than 2 years before the first diagnosis of RA. We changed the referral date into the date of the first diagnosis for RA. Finally, we examined residual confounding due to omission of confounding variables from the adjusted model by including all potential confounders in the model.

### Statistical analysis

We controlled for false discovery rates to compensate for the problem of finding statistically significant results by chance. We considered the effect of statins on the risk of developing RA to be significant at the 0.01 level. We estimated the hazard ratios (HRs) and 99% confidence intervals (CIs) for the risk of developing RA among current, recent and past statin users (versus non-users), using a time-dependent Cox proportional hazards model (SAS 9.2. PHREG procedure).

The following risk factors were considered as potential confounders: use of non-steroidal anti-inflammatory drugs (NSAIDs), aspirin, proton pump inhibitors (PPIs), antibiotics, hormone replacement therapy, antidepressants, anticonvulsants, anti-psychotics, anti-arrhythmic and other lipid-lowering agents within 6 months before the index date [19,29,30,46,47]. In addition, a diagnosis of hypertension, diabetes mellitus, hyperlipidaemia, cardiovascular disease, asthma, inflammatory bowel and thyroid disease, and body mass index (BMI), smoking and alcohol intake (a record of currently smoking or drinking, ex-smoker or -drinker, or never smoked or drank) ever before index date were considered as potential confounders [19,26–31,33,46,48,49]. Next to the matching variables, covariables were included in the final model if they independently changed the β-coefficient for statin use by at least 5%.

We used multiple imputation to address missing data for BMI, smoking and alcohol status. The missing values were imputed by the multiple imputation method using the fully conditional specification method [50]. Twenty imputation were created, analysed and pooled. Results from the complete case and multiple imputation analyses were compared. No difference in the results from both analysed was observed, and multiple imputation analyses are presented.

A descriptive analysis of the pattern of changes in the risk of RA (hazard rates) in current statin users compared to non-users was performed. Non-users included never users but also past and recent users, defined as time = 0. The duration of current use was divided into 10 periods (using every 10^th^ percentile), and hazard rates were calculated within each period. For each period, the risk of RA was plotted against the median time since the first prescription of current statin use and visualised using smoothing spline regression [51], which has been advocated as an alternative to categorical analysis [52].

For all eight sensitivity analyses, we conducted descriptive analyses of the pattern of changes in the risk of RA in current statin users compared to non-users.

Pre-specified subgroup analyses based on the presence of cardiovascular diseases or related risk factors were conducted since previous studies suggested different associations between statin use and the risk of developing RA in patients with a history of cardiovascular diseases, hypertension and diabetes [33,34]. Statins could also have been prescribed to patients with only a diagnosis of diabetes mellitus, or to patients with a low socioeconomic status, or to patients with a family history of cardiovascular disease or to patients with a high-risk ethnicity [53], regardless of their lipid levels, and therefore; we conducted a subgroup analysis in patients with or without a medical history of hyperlipidaemia.

According to previous studies, older or female patients are more likely to experience adverse events of statins than younger or male patients [54,55]. In attempt to examine for this potential bias, we conducted subgroup analyses by age and sex.

We tested for interactions between statin use and age, sex, cardiovascular diseases and related risk factors. Significant interaction terms (P_value_<0.05) were included in the model.

## Results

**Fig 1:**
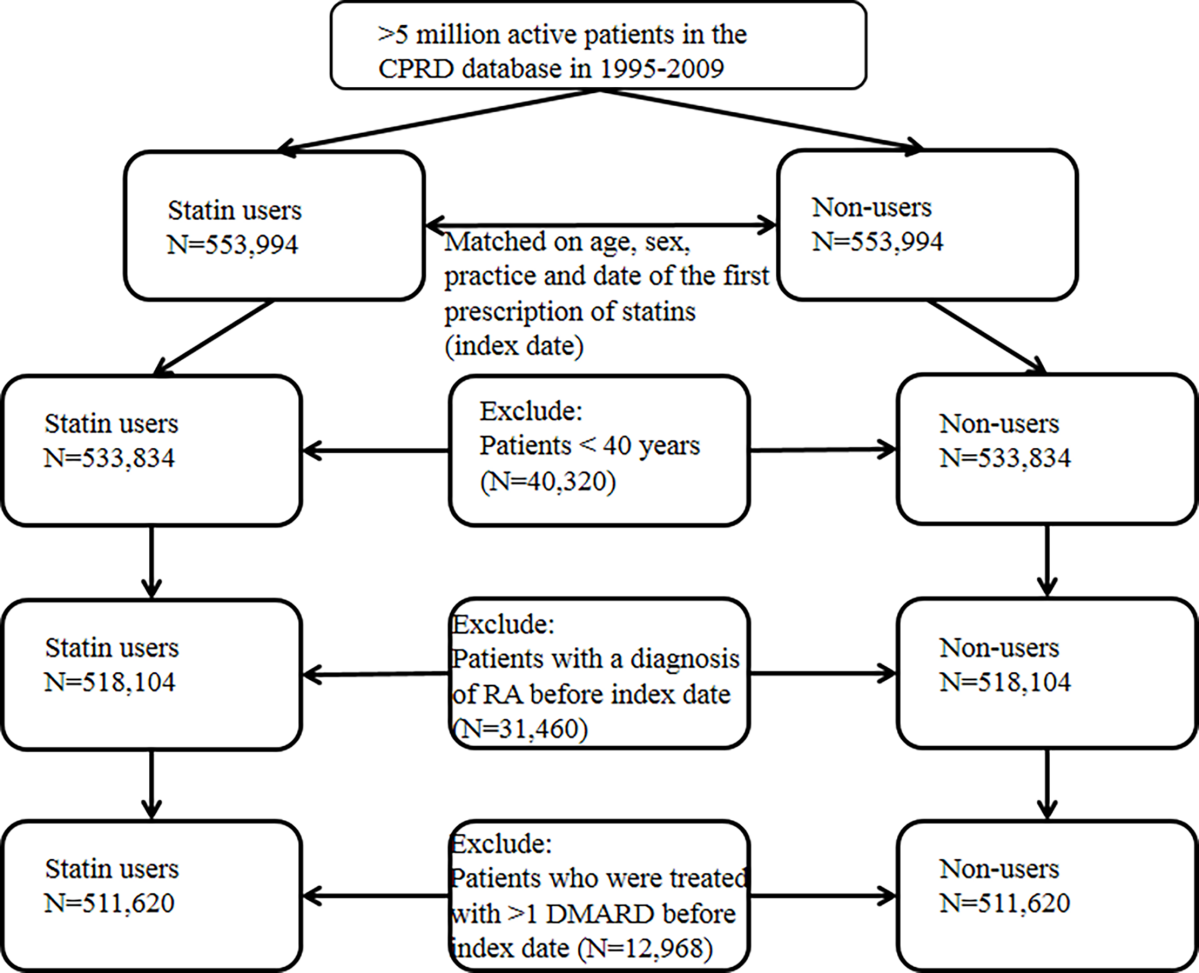
Flow chart study population. Legend: CPRD, Clinical Practice Research Datalink; RA, rheumatoid arthritis; DMARD, disease modifying anti-rheumatic drug.

The study population included 1,107,988 patients. After excluding 40,320 patients who were younger than 40 years, 31,460 patients with a medical history of RA and 12,968 patients with prescriptions of DMARDs before the index date, 511,620 statin users and 511,620 non-users were enrolled in the study (Fig 1).

Due to matching, statin users and non-users had similar distributions of age (statin users: mean age, 63.0 years and non-users: 62.8 years) and sex (statin users and non-users: 48% women). Statin users were more often diagnosed with cardiovascular diseases, hyperlipidaemia, hypertension, diabetes and cerebrovascular events than non-users. Remarkably, smoking was not different between statin users and non-users. Statin users were more likely to have used aspirin, antihypertensive and anti-diabetic agents, NSAIDs, PPIs, antibiotics and antidepressants than non-users (Table 1).

**Table 1:** Baseline characteristics of statin users and non-statin users.

Baseline characteristics	Statin users (n = 511,620)	Non-users (n = 511,620)
Duration of follow-up (years)		
Mean (SD)	3 (2.5)	3 (2.6)
Sex, n (%)		
Women	244,870 (47.9)	244,870 (47.9)
Age (years)		
Mean (SD)	63.0 (12.1)	62.8 (12.5)
BMI (kg/m^2^)		
Mean (SD)	26.9 (8.4)	21.0 (11.6)
Missing	27,760 (5.4)	104,435 (20.4)
Smoking status, n (%)		
Non-smoker	213,102 (41.7)	230,927 (45.1)
Ex-smoker	161,885 (31.6)	109,645 (21.5)
Smoker	114,085 (22.3)	99,340 (19.4)
Missing	22,548 (4.4)	71,708 (14.0)
Drinking status, n (%)		
Non-drinker	63,872 (12.5)	53,309 (10.4)
Ex-drinker	32,104 (6.3)	20,384 (4.0)
Drinker	352,827 (68.9)	317,067 (62.0)
Missing	62,817 (12.3)	120,860 (23.6)
Drug use within previous 6 months, n (%)		
Antihypertensive agents	317,494 (62.1)	121,220 (23.7)
Fibrates	8,436 (1.6)	881 (0.2)
Ezetimibe	1,943 (0.4)	130 (0.03)
Anti-diabetic agents	120,353 (23.5)	18,200 (3.6)
Anti-arrhythmic agents	20,207 (3.9)	11,051 (2.2)
Aspirin	142,209 (27.8)	36,003 (7.0)
NSAIDs	197,750 (38.7)	86,106 (16.8)
Proton pump inhibitors	82,939 (16.2)	46,820 (9.2)
Hormone replacement therapy or oral contraceptives	21,219 (4.1)	20,598 (4.0)
Oral corticosteroids	16,815 (3.3)	14,684 (2.9)
Antibiotics	46,267 (9.0)	35,564 (7.0)
Anticonvulsants	10,648 (2.1)	7,957 (1.6)
Antipsychotics	5,355 (1.0)	6,025 (1.2)
Antidepressants	113,390 (22.2)	93,400 (18.3)
History of disease ever before, n (%)		
Hypertension ^a^	317,523 (72.4)	194,097 (33.2)
Hyperlipidaemia	151,380 (29.6)	12,492 (2.4)
Diabetes^b^	120,681 (23.6)	18,355 (3.6)
Cardiovascular diseases	171,581 (33.5)	46,357 (9.1)
Stroke or TIA	52,336 (10.2)	13,671 (2.7)
Psoriasis	19,719 (3.9)	16,212 (3.2)
Inflammatory bowel disease	5,074 (1.0)	5,034 (1.0)
Cancer	34,369 (6.7)	39,229 (7.7)
Thyroid Disease	52,212 (10.2)	35,735 (7.0)
COPD	20,583 (4.0)	20,283 (4.0)
Asthma	60,252 (11.8)	52,152 (10.2)
Dementia	4,937 (1.0)	8,342 (1.6)
Depression	71,029 (13.9)	48,201 (9.4)

NSAIDs, non-steroidal anti-inflammatory drugs; COPD, Chronic Obstructive Pulmonary Disease; TIA, Transient Ischaemic Attack; SD, standard deviation

^a^ Diagnosis of hypertension or use of antihypertensive agents

^b^ Diagnosis of diabetes mellitus or use of anti-diabetic therapy

The incidence rate for RA is 4.2 per 10,000 person-years. Current users had a risk of developing RA comparable to that of non-users (HR_adjusted (adj)_, 1.06; 99% CI, 0.88 to 1.27) (Table 2). The HR_adj_ for ‘de novo’ users and ‘restart’ users were 1.04; 99% CI, 0.89 to 1.20 and 1.00; 99% CI, 0.86 to 1.50, respectively. Importantly, current statin users who continued the therapy for ≤1 year had a 1.3-fold increased risk of developing RA, (HR_adj_, 1.27; 99% CI, 1.00 to 1.61). Risk of RA was 1.4-fold increased with recent statin use, as compared to non-users (HR_adj_, 1.39; 99% CI, 1.01 to 1.90). No association was found between past statin users and incident RA.

**Fig 2:**
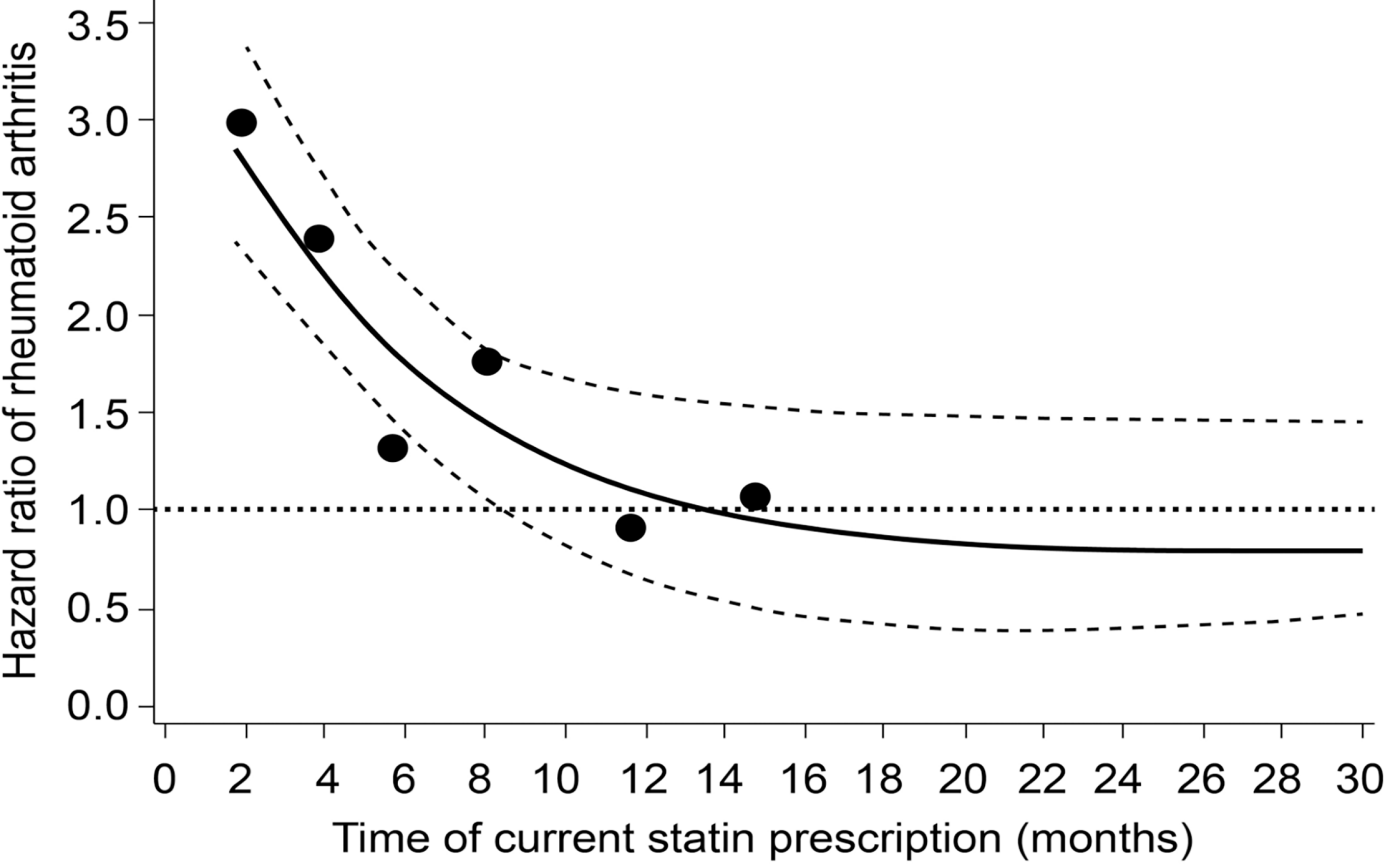
Risk of rheumatoid arthritis in current statin users versus non-users, by time since the first statin prescription. Legend: Solid bold line and circles: adjusted hazard ratios. Dotted lines: 95% confidence bands. Spline regression plot of time since the first prescription of statins and the risk of RA in current statin users vs. matched non-users. HRs are adjusted for confounders as shown in Table 2.

Fig 2 shows that the risk of RA was substantially increased in the first year after the first prescription of statins compared to non-users. The HR_adj_ was 2.93 (99% CI, 2.46 to 3.49) at the start of statin therapy. After one year of statin exposure, the risk of RA declined to baseline level (>1 year: HR_adj_, 0.95; 99% CI, 0.74 to 1.16).

**Table 2:** Risk of rheumatoid arthritis in statin users compared to non-statin users.

	RA (n)	IR ^a^	age- and sex-adjusted HR (99% CI)	fully adjusted HR (99% CI) ^b^
No statin use	579	3.7	1.00	1.00
Past statin use	105	4.9	1.33 (1.08 to 1.64)	1.18 (0.88 to 1.57)
Recent statin use	101	5.6	1.60 (1.27 to 2.02)	1.39 (1.01 to 1.90)
Current statin use	837	4.3	1.24 (1.10 to 1.38)	1.06 (0.88 to 1.27)
≤ 1 year	386	11.5	1.47 (1.25 to 1.73)	1.27 (1.00 to 1.61)
> 1 year	451	2.8	1.15 (1.02 to 1.31)	0.98 (0.80 to 1.19)
‘de novo’ statin use	464	4.1	1.16 (1.02 to 1.31)	1.04 (0.89 to 1.20)
‘restart’ statin use	373	4.7	1.34 (1.17 to 1.54)	1.00 (0.86 to 1.50)

RA, rheumatoid arthritis; IR, incidence rate (per 10 000 person-years); HR, hazard ratio; CI, confidence interval

^a^ Incidence rate is calculated for each recency of statin use by dividing the number of events by the person time within each given recency of use.

^b^Adjusted for age, sex, practice, smoking, cardiovascular diseases, hyperlipidaemia, hypertension, diabetes and use of non-steroid anti-inflammatory drugs.

In Table 3, we present several potential risk factors that may have influenced the risk of developing RA after statin exposure. We observed a tendency towards an increased risk of developing RA in women and patients without a medical record of hyperlipidaemia, hypertension and diabetes who currently used statins for less than one year, or who were recent users. However, for none of these potential risk factors, the interaction term did reach significance. No effect modifiers for the association between current, recent and past statin exposure and incident RA were found.

**Table 3:** Risk of rheumatoid arthritis risk in statin users vs. non-statin users according to different populations.

			adjusted HR (99% CI) ^b^				
	RA	IR ^a^	Past statin use	Recent statin use	Current statin use	Current statin use	Current statin use
	(n)					≤ 1 year	> 1 year
By age, y							
40–50	131	2.6	2.23 (0.95 to 5.18)	1.57 (0.49 to 5.00)	1.29 (0.63 to 2.65)	1.06 (0.43 to 2.64)	1.39 (0.66 to 2.93)
51–60	475	4.1	1.11 (0.66 to 1.90)	1.60 (0.91 to 2.82)	0.94 (0.66 to 1.33)	1.28 (0.82 to 2.02)	0.80 (0.55 to 1.18)
61–80	938	5.1	1.08 (0.73 to 1.60)	1.19 (0.77 to 1.84)	1.01 (0.79 to 1.28)	1.21 (0.89 to 1.65)	0.93 (0.72 to 1.20)
>80	78	2.0	1.21 (0.38 to 3.79)	1.49 (0.43 to 5.13)	0.93 (0.43 to 2.02)	1.06 (0.40 to 2.76)	0.86 (0.36 to 2.05)
By sex							
Women	1,021	5.4	1.19 (0.83 to 1.69)	1.68 (1.14 to 2.50)	1.17 (0.93 to 1.47)	1.49 (1.09 to 2.02)	1.06 (0.83 to 1.36)
Men	601	3.0	1.19 (0.74 to 1.93)	0.99 (0.58 to 1.71)	0.89 (0.66 to 1.21)	0.99 (0.68 to 1.44)	0.85 (0.61 to 1.18)
By any previous history of disease							
No previous cardiovascular disease	1,138	3.9	1.24 (0.90 to 1.72)	1.35 (0.91 to 1.99)	1.00 (0.80 to 1.24)	1.19 (0.90 to 1.57)	0.91 (0.72 to 1.16)
Previous cardiovascular disease	484	4.8	0.96 (0.50 to 1.83)	1.32 (0.72 to 2.46)	1.06 (0.68 to 1.64	1.28 (0.77 to 2.15)	0.99 (0.63 to 1.55)
No previous cardiovascular risk factor^c^	649	4.0	1.18 (0.72 to 1.93)	1.52 (0.87 to 2.63)	1.04 (0.77 to 1.39)	1.37 (0.93 to 2.01)	0.84 (0.58 to 1.21)
Previous cardiovascular risk factor	973	4.3	1.05 (0.73 to 1.53)	1.19 (0.87 to 1.61)	0.94 (0.73 to 1.21)	1.09 (0.79 to 1.49)	0.89 (0.69 to 1.16)
No previous hyperlipidaemia	1,254	4.1	1.11 (0.79 to 1.57)	1.59 (1.12 to 2.28)	1.05 (0.86 to 1.29)	1.24 (1.01 to 1.57)	0.98 (0.79 to 1.23)
Previous hyperlipidaemia	368	4.5	1.81 (0.69 to 4.70)	1.32 (0.47 to 3.71)	1.48 (0.61 to 3.58)	1.96 (0.77 to 5.00)	1.34 (0.55 to 3.28)
No previous hypertension	857	4.1	1.25 (0.84 to 1.85)	1.29 (0.81 to 2.05)	1.12 (0.87 to 1.44)	1.50 (1.08 to 2.07)	0.94 (0.70 to 1.26)
Previous hypertension	765	4.3	0.99 (0.64 to 1.51)	1.29 (0.91 to 1.81)	0.88 (0.67 to 1.17)	0.96 (0.67 to 1.36)	0.86 (0.64 to 1.15)
No previous diabetes	1,394	4.2	1.16 (0.86 to 1.58)	1.39 (0.98 to 1.98)	1.07 (0.88 to 1.30)	1.31 (1.02 to 1.70)	0.97 (0.78 to 1.20)
Previous diabetes	228	4.1	1.10 (0.41 to 2.94)	1.14 (0.44 to 2.97)	0.89 (0.41 to 1.91)	0.92 (0.40 to 2.13)	0.88 (0.40 to 1.91)

RA, rheumatoid arthritis; IR, incidence rate (per 10,000 person-years); HR, hazard ratio; CI, confidence interval

^a^ Incidence rate is calculated for each recency of statin use by dividing the number of events by the person time within each given recency of use.

^b^Adjusted for confounders as shown in Table 2.

^c^Cardiovascular risk factor included previous hyperlipidaemia, hypertension and diabetes

### Sensitivity analyses

Performing different sensitivity analyses did alter our findings slightly. The sensitivity analysis where we excluded the first year after initiation of statin treatment showed that current use, who were divided by the duration of their therapy was not associated with a risk of RA. All other sensitivity analyses showed similar results as the main analysis, although they were slightly attenuated. When we depicted the patterns of changes in the risk of RA during current statin use of all the sensitivity analyses, no differences in the patterns of all the sensitivity analyses were observed. The results of the eight sensitivity analyses are presented online (S2 Table).

## Discussion

This study demonstrated a 1.3-fold increased risk of developing RA during the first year of statin use. The risk of developing RA was increased shortly after the first prescription of statins and then gradually decreased to baseline level. In recent users, statin use was associated with incident RA whereas in past users no such effect was found.

Two population-based cohort studies showed no association between statin use and the risk of developing RA [28,29]. Furthermore, in a nested case-control study of 313 incident RA patients and 1,252 matched controls, no association between current statin use and incident RA was found [26]. The same study showed, however, in a subsample of only patients with hyperlipidaemia a decreased risk of developing RA in current users [26]. We found no association between current statin use and incident RA in patients with hyperlipidaemia, although the sample size was too small to draw a definite conclusion. However, the differences in findings may be explained by the difference in defining statin exposure. Jick et al. defined current statin use as receiving two prescriptions in the year prior to the first diagnosis of RA [26] whereas we defined it as the time from the date of a prescription until three months after its expected duration of use (time-dependent variable).

In a population-based cohort study of 211,627 new statin users, statin use was associated with a reduction in the risk of developing RA. This effect was only present for those who used statins for more than one year [27]. In contrast, we found no association between statin use and incident RA in current users who continued statin therapy for more than one year. Another population-based cohort study of 528,654 new statin users showed that high-intensity statin treatment was associated with a reduced risk of incident RA in comparison to low-intensity statins [31]. In our study we did not observe the potential beneficial effects of statins. The difference between our study and Tascilar et al. may be explained by the selection of the reference group [31]. In a propensity score matched cohort study of 6,956 pairs of statin users and non-users, statin use was associated with a lower risk of connective tissue disease (CTD) [30]. The possible protective effect of statins was not observed in our study. The discrepancies may be partially explained by the difference in defining statin exposure and defining RA. We classified statin exposure by the recency of use, and modelled it as a time-dependent variable. Chodick et al. and Tascilar et al., however, defined statin exposure as the proportion of follow-up days covered with statins [27,31], whereas Schmidt et al. defined statin use as receiving at least a 90-day supply at baseline [30]. Also, our definition of RA may have been more specific than the ones used by other three studies [27,30,31]. We verified patients’ electronic records with at least one prescription of a DMARD after the diagnosis of RA. Tascilar et al. used the same approach of defining RA as we did, but on top of that, they included patients with two electronic records for RA at least three months apart [31]. In the study by Chodick et al., patients were included when they had a diagnostic code for RA with or without the use of DMARDs [27], whereas Schmidt et al. included patients with CTD, including RA [30]. Using a more specific definition of RA may have influenced the association between statin use and incident RA.

Another explanation for the conflicting results between these six observational studies [26–31] and ours may be the selection of the non-users. In two other studies non-users were also selected at the date of the first prescription of statins, although these non-users were matched or adjusted for propensity scores, including age, sex and other baseline characteristics [29,30]. Smeeth et al. excluded [29], however, the first year of every patient following the initiation of statin treatment. In another study, non-users were again selected at the date of the first prescription of statins, however, no matching was conducted. Instead, the analyses were adjusted for age, BMI, ethnicity, smoking and hypothyroidism in men and women separately [28]. The other three studies selected the reference group as patients who were non-persistent statin users [27], or patients who were exposed to low-intensity statin treatment [31], or controls as patients without a diagnosis of RA at the date of the first diagnosis of RA [26]. We believe that the selection of the reference group (non-users) may have influenced the study results. To address the possible differences in baseline characteristics between statin users and non-users, we did consider the use of a propensity-matched cohort of non-users. However, recent research has found that propensity matching does not always improve the adjustment of measured confounding [56]. Besides this, unmeasured confounding is not addressed by propensity score matching [56]. We used a study population comprising a matched sample of the general population (i.e. matched on age, sex, general practice and calendar time) to reduce bias due to differences between the statin users and non-users. In addition, we adjusted our models for potential confounders. Further, we conducted a sensitivity analysis where we included all potential confounders in the model. The effect estimates were similar to those presented in the main analysis. By using various methods for handling the potential differences between the statin users and non-users, we believe that we have selected a representable reference group. Furthermore, the incidence rate of RA in the non-users of our study is almost in line with the incidence rate of RA as presented in other studies [57,58]. Therefore, we suggest that the non-users may be a good reflection of the general population.

In our previous study [19], we found an increased risk of developing RA within six months of statin use, which is in line with the results of the present study. In this study, we found that the risk of developing RA disappeared after one year. Statins may accelerate the onset of RA in patients susceptible to develop RA as previously demonstrated in an animal model of arthritis [20]. In line with this hypothesis, the majority of cases of statin-associated lupus-like syndrome developed this syndrome within one year after starting statin therapy [16]. However, in patients not prone to develop RA, statins are probably safe and well tolerated, even after prolonged use. In this case, it should be stressed that patients with a high (>20% risk) 10-year risk of cardiovascular disease or intermediate (>10% risk) 10-year risk should continue statin treatment. In a recent study, it has been shown that statin cessation after media debates regarding safety could result in increased statin cessation and at least 2,173 excess cardiovascular events over 10 years in the UK [59].

RA may have been present and not well documented before the start of statins, which may have introduced bias (protopathic) in this study. In both our studies, we defined the onset date of RA by the first record or specialist referral. Unfortunately, the onset date of symptoms is unknown in our studies. One study reported a median time between onset of symptoms to diagnosis of RA of approximately 36 weeks (range: 4 weeks to >10 years) [60]. As two population-based studies considered a lag-time of one year between statin use and incident RA [27,29], we performed a sensitivity analysis where we excluded the first year following the initiation of statin treatment. We found no increased risk of RA in the first year after the excluded year. However, the descriptive analysis of this sensitivity analysis showed a similar pattern of risks of RA during statin use but was slightly attenuated.

Importantly, cardiovascular risk factors (e.g. hyperlipidaemia) may have influenced the association between statin use and incident RA. Several studies have demonstrated an unfavourable lipid profile in patients with RA [32,37]. Hyperlipidaemia may induce leukocyte activation and possibly complement activation [61–63], which may result in an earlier diagnosis of RA in patients prone to develop RA. When we conducted subgroup analyses in patients with and without a medical record for hyperlipidaemia, patients with hyperlipidaemia showed high risks of developing RA with current, recent and past statin use, although the sample sizes were too small for conclusions.

The mechanism and time course by which statins may facilitate RA are unknown. According to one review, the mean time of exposure before disease onset ranges from one month to six years [16]. Despite the unknown time course, it has been suggested that statins may promote a shift in Th1/Th2 balance [16,22] or affect regulatory T cells [13,64,65], or lead to unstable regulatory T cells in the periphery [66,67], and thus may promote autoimmunity. Based on these findings, we hypothesise that statins do not themselves cause autoimmunity but they may promote a pre-existing autoimmune-prone condition to progress towards a clinical manifest disease such as RA. Another possible hypothesis is that the self-tolerance is lost due to persistence of infectious agents in individuals who were treated with statins. Since statins may reduce Th1 responses [9], infectious agents may not be cleared as efficiently as under normal circumstances [68].

Strengths of this study include its large sample size, representativeness of the population, completeness of follow-up and information on matched non-users, and detailed information on confounders, such as smoking status was available [35,48]. Further, data are prospectively collected in the CPRD, and thus not subjected to recall bias.

Some drawbacks of our study should be considered.

First, the information about statin exposure was based on prescription data rather than on actual drug use, which could have resulted in an overestimation of statin use.

Second, although we have used a relatively specific diagnosis of RA, we had limited information on rheumatoid factor and anti-cyclic citrullinated peptide antibodies [38]. In this study, we have used the diagnostic algorithm as postulated by Thomas et al [44]. The proposed diagnostic algorithm resulted in a diagnostic specificity of 96% and sensitivity of 78% [44]. By applying this diagnostic algorithm, we believe we have used a relatively accurate diagnosis of RA. Furthermore, we performed three sensitivity analyses regarding the definition of RA. All of them consistently showed similar results.

Third, no data on dietary intake, physical activity, and limited data were available on other examinations such as lipid, blood pressure and glucose levels, and inflammatory markers (e.g. C-reactive protein), which may be important confounders. Especially, in the subgroup analyses based on the cardiovascular risk factors, lack of clinical data may have affected our results. It is likely that we have included patients with high lipid, glucose or high blood pressure levels in the group of patients without a medical history of hyperlipidaemia, hypertension or diabetes.

Fourth, our study cannot be considered as a definitive study. A randomised trial evaluating the effects of statins on the development of RA would be ideal, but that this is unlikely to occur due to infrequency of RA and thus need for a very large study.

Fifth, the increased risk of RA in the first year after the initiation of statin therapy may be explained by ascertainment bias, as some patients initiating statin therapy may experience myalgia or other muscle-related adverse effects [69]; they may tend to visit their GP more often, be more likely to be referred to a rheumatologist and may have been more carefully examined (blood tests) [28,70], therefore; these patients may be more likely to be diagnosed with RA than non-users. In the sensitivity analysis where we excluded the first year following the initiation of statin treatment, we found no increased risk of RA in the first year of statin use after the excluded year; nor did we observe an increased risk of RA in recent and past statin users. Therefore, we believe that ascertainment bias may have influenced our findings, and therefore; it is more than likely that the association between statin use and the increased risk of developing RA in the first year after initiating statin treatment, is not causal.

## Conclusions

To our knowledge, this is the first study evaluating risks of RA in statin users over time. In patients who use statins, the risk of RA is substantially increased in the first year after initiation of statins and then diminishes to baseline, suggesting an association between statin use and an increased risk of RA in the first year after initiating statin treatment. Our finding may suggest that statins can accelerate disease onset in patients susceptible to develop RA, but in other patients, statins are probably safe and well tolerated, even after prolonged use. Another explanation for this increased risk of RA shortly after starting statins is ascertainment bias with increased diagnostic monitoring around the time of initiation of statin therapy. Although more research is needed, this study supports our previous finding showing an increased risk of developing RA shortly after starting statin treatment.

## Supporting information

S1 Fig Study design sensitivity analyses. Index date: the date of the first prescription
Sensitivity analysis 1: exclude the first year of every patient following the initiation of statin treatment (index date), thereby excluding the events of RA
Sensitivity analysis 2: change the date of the first diagnosis of RA to exactly one year before this date.
(TIFF)

S1 Table All observational studies showing the risk of developing rheumatoid arthritis with statin use. (DOCX)

S2 Table Several sensitivity analyses to test the robustness of our findings. Legend:
RA, rheumatoid arthritis; IR, incidence rate (per 10,000 person-years); HR, hazard ratio; CI, confidence interval
^a^Incidence rate is calculated for each sensitivity analysis by dividing the number of events by the person time within each given recency of use.
^b^Adjusted for age, sex, smoking, cardiovascular diseases, hyperlipidaemia, hypertension, diabetes and the use of NSAIDs.
(DOCX)
