# Supplementary figures and images for: Pattern of risks of rheumatoid arthritis among patients using statins: A cohort study with the clinical practice research datalink
Source: PLoS One. 2018 Feb 23;13(2):e0193297. doi: 10.1371/journal.pone.0193297 (PMC5825093; doi:10.1371/journal.pone.0193297)

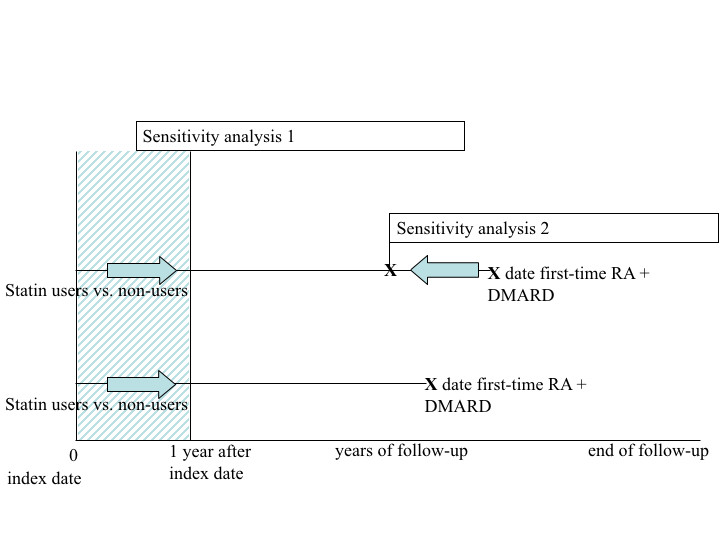

Supplement: S1 Fig — Index date: the date of the first prescription Sensitivity analysis 1: exclude the first year of every patient following the initiation of statin treatment (index date), thereby excluding the events of RA Sensitivity analysis 2: change the date of the first diagnosis of RA to exactly one year before this date. (TIFF) [file pone.0193297.s001.tiff]
