# Supplementary material for: Pattern of risks of rheumatoid arthritis among patients using statins: A cohort study with the clinical practice research datalink
Source: PLoS One. 2018 Feb 23;13(2):e0193297. doi: 10.1371/journal.pone.0193297 (PMC5825093; doi:10.1371/journal.pone.0193297)
Supplement: S1 Table — (DOCX) [file pone.0193297.s002.docx]

**S1 Table.** All observational studies showing the risk of developing rheumatoid arthritis with statin use

| Observational studies of statin-associated RA | Smeeth et al., 2008^29^ | Jick et al., 2009^26^ | Hippisley-Cox et al., 2010^28^ | Chodick et al., 2010^27^ | Schmidt et al., 2013^30^ | Tascilar et al., 2016^31^ |
| --- | --- | --- | --- | --- | --- | --- |
| Data source | UK Health Improvement Network Database (THIN) | General Practice Research Database (GPRD) | QResearch Database | Maccabi Healthcare Service (MHS) | San Antonio area military health care system | UK Clinical Practice Research Datalink (CPRD) |
| Study design | Retrospective cohort | Nested case-control | Prospective cohort | Retrospective cohort | Retrospective cohort | Nested case-control |
| Study population | ∙All patients registered with a GP between Jan 1995 - Dec 2006  ∙Comprising EMR for 5.5 million patients derived from 303 practices | ∙All patients registered with a GP between Jan 1, 1992 – Dec 31, 2001  ∙Since 1987, ≥ 5 million residents in the UK have been enrolled | ∙All practices in England and Wales using the computer based Egton Medical Information System (EMIS)  ∙All patients registered with the practices between Jan 1, 2002 – June 30, 2008 | ∙All patients who were continuously enrolled in the Israel Health Maintenance Organisation (HMO) from 1995-1998 were included in the cohort (Jan 1, 1998 – July 1 2007)  ∙Comprising 1.8 million people in the HMO | ∙All patients who were enrolled as Tricare Prime or Tricare Plus in the San Antonio are military health care system between Oct 1, 2003- Mar 5, 2010  ∙Study period was divided into baseline period (Oct 1, 2003-Sept 30, 2005) for describing the baseline characteristics and follow-up period (Oct 1, 2005-Mar 5, 2010) for identifying connective tissue diseases | ∙All new statin users from 623 general practices that contributed data to the CPRD between Jan 1, 1997 and Dec 31, 2009 |
| Exposure to statins | ∙All patients aged 40-80 years who received their first prescription for a statin on or after Jan 1, 1995  ∙Date of the first prescription of statins is defined as index date | ∙All patients aged 40-89 years with at least 1 prescription for a statin at any time  ∙Current statin use was defined as receipt of at least two prescriptions within 1 year preceding the first-time diagnosis of RA  ∙All other receipt of statins was considered past use | ∙All new users of statins aged 30-84 years during the study period | ∙All patients aged ≥18 years who had at least 1 dispensed prescription of statins during the study period  ∙Date of the first dispensed statin is defined as index date | ∙All patients aged 30-85 years who received a statin prescription of at least 90-day supply between Oct 1, 2004-Sept 30, 2005 | ∙All new statin users of at least 40 years of age  ∙Date of the first prescription of statins is defined as cohort entry date  ∙The number of days supplied (NDS) with each statin prescription was calculated by dividing the prescribed number of units by the daily number of units  ∙Statin intensity (based on the percent reduction in low density lipoprotein) was weighted by the NDS between the date of the first statin prescription and index date for each patient  ∙The duration-weighted average statin intensity was ranked in the following quintiles: ≤0.93, >0.93-1, >1-1.09, >1.09-1.2, >1.2 |
| Control group | ∙Up to 5 non-users of statins were matched to each user on sex and age within 5 years | ∙Randomly selected up to 4 controls, i.e. people without a diagnosis of RA  ∙Matched by age (within 1 year), sex, calendar time (using the same date as the first-time diagnosis of RA), practice and number of years of previous recorded history | ∙Non-use of statins during the study period | ∙<20% proportion of days covered with statins | ∙Did not receive a statin at any time during the study | ∙Randomly selected up to 10 controls, i.e. people without a diagnosis of RA  ∙Matched by age (within 5 years), sex, calendar year (using the same year as the first-time diagnosis of RA) |
| Medical history | ∙At least one year before start statin treatment | - | ∙At least one year registered with the general practice when the date of the study started | ∙Patients who did not receive a statin prescription at least 3 year prior to the index date | ∙At least one year before start statin treatment (start date baseline period Oct 1, 2003 and start date statin prescription Oct 1, 2004) | ∙At least one year before the first statin prescription |
| Definition RA | ∙First time diagnosis of RA | ∙First time diagnosis of RA  ∙Included cases with a stated diagnosis of RA and/or a referral to outpatient specialist or inpatient hospitalization and use of DMARDs | ∙First time diagnosis of RA | ∙First time diagnosis of RA | ∙The occurrence of 3 separate codes for connective tissue disorder, including RA, SLE, dermatomyositis, polymyositis, polymyalgia rheumatica, sicca syndrome, keratoconjuctivitis sicca, Sjögren’s disease and connective tissue disorder (unspecified) during the follow-up period | ∙Date of the first fulfillment of the case definition of RA  ∙An algorithm including 2 sets of qualifiers with 2 qualifiers each: 1) one diagnostic code for RA and one prescription for a DMARD; 2) appearance of 2 diagnostic codes for RA at least 3 months apart |
| Shifting the date of the first diagnosis of RA | - | ∙Exactly one year prior to the date of the first RA diagnosis (index date) | - | - | - | ∙Three years prior to the date of the first fulfillment of the case definition (RA) (index date) |
| Considering a lag-time | ∙Cases of RA in the first year after statin initiation were excluded | - | - | ∙Cases of RA in the first year after statin initiation were excluded | - | ∙Cases of RA in the first year after statin initiation were excluded |
| Time-dependent analysis | - | - | ∙To determine the risk of RA within 1 year, 1-3 years, 3-5 years and ≥5 years of taking statins | - | - | - |
| Confounders | ∙Propensity score* at index date, year initiation of statin treatment, first diagnosis of the following post-index date: diabetes, cerebrovascular disease, coronary heart disease, peripheral vascular disease, other atheroma, atrial fibrillation, heart failure, hyperlipidaemia, hypertension, other circulatory disease, cancer, dementia, first use of the following post-index date: aspririn, nitrates, fibrates, β-blockers, CCB, potassium channel activators, diuretics, positive inotropes, anticoagulants, anti-hypertensive drugs, or other cardiovascular drugs | ∙Smoking before index date | ∙Age, BMI, ethnicity, smoking, hypothyroidism at start study | ∙Age, sex, socioeconomic level, utilization of healthcare services in the year before the index date | ∙In the propensity score matched cohort: no correction for confounders  ∙In the unmatched cohort: adjusted for all covariates which were included in the propensity score model | ∙Smoking status, total cholesterol levels, obesity, history of cardiovascular disease, coexistent autoimmune diseases, hypothyroidism and persistence with treatment before/at start study |
| Propensity score | ∙Adjusted for propensity score*  ∙Propensity score is based on factors that may influence statin prescribing | - | - | - | ∙Matching on propensity score** | - |
| Subgroup analysis | - | ∙Hyperlipidaemia, type of statins | ∙Sex, type of statins | ∙Age categories, sex, baseline LDL levels and efficacy of initial statin therapy | - | ∙Recency of statin use |

RA, rheumatoid arthritis; GP, general practitioner; EMR, electronical medical records; UK, United Kingdom; DMARDs, disease-modifying anti-rheumatoid drugs; BMI, body mass index; CCB, calcium channel blockers; LDL, low-density lipoprotein

*included covariates in the propensity score model: body mass index, socioeconomic status, consultation rate, prescribing rate, smoking status, drinking habits, diabetes, coronary heart disease, cerebrovascular disease, peripheral vascular disease, other atheroma, other circulatory disease, dementia, cancer, atrial fibrillation, heart failure, recent hepatic disease, recent renal disease, thyroid disease, hyperlipidaemia, hypertension, recent use of hormone replacement therapy, anti-psychotics, antidepressants, steroids (oral or inhaled), fibrates, cytochrome P450 3A4 inhibitors, any prior use of non-statin or fibrate lipid-lowering medication, nitrates, aspirin, β-blockers, calcium channel blockers, potassium channel activators, diuretics, positive inotropes, anticoagulants, anti-hypertensive drugs, or other cardiovascular drugs.

**included covariates in the propensity score model: age, sex, acute myocardial infarction, congestive heart failure, peripheral vascular disease, cerebrovascular disease, dementia, chronic obstructive pulmonary diseases, rheumatologic diseases, peptic ulcer disease, mild liver disease, diabetes mellitus, diabetes mellitus complications, hemiplegia/paraplegia, renal disease, malignancy, liver disease (moderate/severe), metastatic neoplasm, HIV, illicit drug use, alcohol abuse/dependence, smoker, total Charlson co-morbidity index, number of outpatient medical encounters and inpatient admissions during each of the baseline period and the follow-up period, and the use of β-blockers, calcium channel blockers, diuretics, non-statin lipid-lowering drugs, angiotensin receptor blockers/ angiotensin converting enzyme inhibitors, oral hypoglycemic, cytochrome P450, aspirin, non-steroidal anti-inflammatory drugs, selective serotonin reuptake inhibitors, systemic corticosteroids, anti-psychotics, sedatives, tricyclic antidepressants.
