# Supplementary material for: Pattern of risks of rheumatoid arthritis among patients using statins: A cohort study with the clinical practice research datalink
Source: PLoS One. 2018 Feb 23;13(2):e0193297. doi: 10.1371/journal.pone.0193297 (PMC5825093; doi:10.1371/journal.pone.0193297)
Supplement: S2 Table — Legend: RA, rheumatoid arthritis; IR, incidence rate (per 10,000 person-years); HR, hazard ratio; CI, confidence interval aIncidence rate is calculated for each sensitivity analysis by dividing the number of events by the person time within each given recency of use. bAdjusted for age, sex, smoking, cardiovascular diseases, hyperlipidaemia, hypertension, diabetes and the use of NSAIDs. (DOCX) [file pone.0193297.s003.docx]

**S2 Table.** **Several sensitivity analyses to test the robustness of our findings**

|  |  |  | adjusted HR (99% CI) ^b^ | | | | |
| --- | --- | --- | --- | --- | --- | --- | --- |
| Sensitivity analyses | RA | IR ^a^ | Past statin use | Recent statin use | Current statin use | Current statin use | Current statin use |
|  | (n) |  |  |  |  | ≤ 1 year | > 1 year |
| 1.Restrict to RA patients referred to a rheumatologist or received at least one prescription of a DMARD after the first-time diagnosis of RA | 1,977 | 5.1 | 1.20 (0.98 – 1.46) | 1.34 (1.08 – 1.67) | 1.04 (0.92 – 1.18) | 1.18 (1.00 – 1.39) | 0.98 (0.86 – 1.12) |
| 2.Restrict to RA patients referred to a rheumatologist or received at least one prescription of a DMARD and/or received at least two prescriptions of corticosteroids after the first-time diagnosis of RA | 2,162 | 5.5 | 1.18 (0.98 – 1.42) | 1.29 (1.04 – 1.59) | 1.04 (0.93 – 1.18) | 1.16 (0.99 – 1.35) | 1.00 (0.88 – 1.13) |
| 3.Restrict to RA patients with at least one medical record after the first-time diagnosis of RA | 1,171 | 3.0 | 1.22 (1.05 – 1.43) | 1.08 (0.90 – 1.31) | 0.98 (0.88 – 1.14) | 1.10 (0.95 – 1.23) | 0.94 (0.84 – 1.05) |
| 4.Restrict to RA patients who received at least one prescription of a DMARD after the first-time diagnosis of RA in a time span of two years after the first-time diagnosis | 1,530 | 3.9 | 1.16 (0.93 – 1.45) | 1.29 (1.00 – 1.66) | 1.01 (0.88 – 1.17) | 1.17 (1.00 – 1.41) | 0.95 (0.82 – 1.11) |
| 5.Exclude the first year after initiation of statin treatment | 1,308 | 3.9 | 1.16 (0.92 – 1.45) | 1.12 (0.85 – 1.48) | 0.90 (0.76 – 1.06) | 0.82 (0.58 – 1.08) | 0.99 (0.82 – 1.21) |
| 6.Shift the event (RA) date exactly one year before the date of the first-time diagnosis of RA | 1,258 | 3.2 | 1.25 (0.98 – 1.60) | 1.04 (0.77 – 1.40) | 1.11 (0.94 – 1.30) | 1.15 (0.99 – 1.31) | 1.13 (0.95 – 1.33) |
| 7.Change the event (RA) date when the referral to a rheumatologist was more than 2 years before the first-time diagnosis of RA | 1,395 | 3.8 | 1.15 (0.94 – 1.43) | 1.26 (0.97 – 1.76) | 1.02 (0.87 – 1.21) | 1.14 (0.98 – 1.29) | 0.97 (0.83 – 1.18) |
| 8. Include all potential confounding variables in the model to examine residual confounding | 1,622 | 4.2 | 1.17 (0.88 – 1.55) | 1.38 (1.01 – 1.90) | 1.07 (0.89 – 1.29) | 1.27 (1.00 – 1.62) | 0.99 (0.81 – 1.21) |
|  |  |  |  |  |  |  |  |

RA, rheumatoid arthritis; IR, incidence rate (per 10,000 person-years); HR, hazard ratio; CI, confidence interval

^a^Incidence rate is calculated for each sensitivity analysis by dividing the number of events by the person time within each given recency of use.

^b^Adjusted for age, sex, smoking, cardiovascular diseases, hyperlipidaemia, hypertension, diabetes and the use of NSAIDs.
